# Deformation characteristics test and mechanism of arbor taproot soil complex in rainforests

**DOI:** 10.1038/s41598-023-32304-1

**Published:** 2023-04-07

**Authors:** Peng Du, Dequan Zhou, Xiaoling Liu, Yujie Feng

**Affiliations:** 1 Changsha University of Science and Technology, National Engineering Laboratory of Highway Maintenance Technology, Changsha, 410114 Hunan China; 2 Haikou University of Economics, Yaha School of Built Environment, Haikou, 571127 Hainan China; 3 Hainan Communications Planning Surveying and Designing Institute, Haikou, 570206 Hainan China

**Keywords:** Civil engineering, Environmental impact

## Abstract

This study performed large-scale single shear tests on Haikou red clay and arbor taproot to explore the anti-sliding effect and deformation characteristics of rainforest arbor roots under a shallow landslide. The law of root deformation and the root–soil interaction mechanism were revealed. The results indicated the significant reinforcing effect of arbor roots on the shear strength and ductility of soil, which increased with the decrease of normal stress. The soil reinforcement mechanism of arbor roots was attributed to their friction and retaining effects through an analysis of the movement of soil particles and the deformation pattern of roots during the shear process. The root morphology of arbors under shear failure could be described using an exponential function. Consequently, an advanced Wu model which better reflected the stress state and deformation of roots was proposed based on the concept of curve segment superposition. The results are believed to a reliable experimental and theoretical basis for the in-depth study of soil consolidation and sliding resistance effects of arbor roots, and further lay a foundation for the slope protection by arbor roots.

## Introduction

With the development of the national economy, the multitude of infrastructure constructions have severely affected the environment. Artificial slope-cutting has produced several artificial slopes, which have exacerbated soil erosion and caused serious damage to the ecological environment. The traditional engineering for slope protection mostly adopts reinforced concretes for strengthening; however, it is expensive with a complex construction process, poor durability, and a monotonous landscape effect. Ecological slope protection is a comprehensive technology for slope protection, involving only plants or the combination of engineering and plants, which can aid in the realization of both engineering construction and ecological protection. Consequently, it has become a research hotspot of numerous scholars worldwide.

In recent years, scholars have conducted several studies on the soil consolidation effect of plant roots. Indoor or field tests have confirmed the ability of plant roots in significantly improving the shear strength and ductility of soil^1–9^. These research results have shown that the natural attributes of roots considerably affected the soil consolidation effect, such as their species^10–15^, growth age^16–19^, and root morphology^20–24^. In general, the content of roots was found to be positively correlated with the shear strength of root–soil complex^25–28^, while Yang et al.^29^, Li et al.^30^, Liao et al.^31^, and Wang et al.^32^ discovered through tests that there was an optimal roots content for their strengthening effect on soil. In addition, Deng et al.^33^, Gai et al.^34^, and Feng et al.^35^ revealed that the distribution and location of roots also significantly influenced the soil consolidation effect. Most studies have mainly focused on the soil consolidation by herbs and shrubs; however, studies on large-scale shear tests of arbor roots are scarce. Moreover, restricted by experimental instruments and detection methods, reports on the morphological characteristics of roots and the mechanism of root–soil interaction are rare. Wen et al.^36^ and Zhao et al.^37^ conducted a large-scale direct shear test of root–soil complex with different root distribution methods in case of a Haikou rubber tree taproot. The root deformation was realized by connecting PVC high-strength fiber with the root; however, the test resulted in the artificial setting of the surface of shear failure, which was unable to truly reflect the shear deformation of soil and root in the shear process.

To explore the theory and mechanism of roots consolidating soil, Waldron^38^, Wu et al.^39^, and Gray and Ohashi^40^ jointly established the root–soil consolidation model (Wu model) based on the Mohr-Coulomb strength theory. However, this model presumed that all roots were pulled off simultaneously, resulting in generally larger shear strength. Pollen and Simon^41^ constructed a fiber bundle model (FBM) for describing root consolidation. It was based on the progressive fracture of roots during the process of soil shearing. Although the calculation results were closer to the direct shear test than the Wu model, the root strength distribution was determined according to the probability of field measurements, which was not considered reliable. Sui and Yi^42^ adopted fracture mechanics and functional principles to build a mechanical model of root soil consolidation. The direct shear test data results indicated a high calculation accuracy; however, the key fracture toughness parameters in the model were related to both the root diameter and growth age. Moreover, the model still needs to be further validated and improved. The above typical mechanical models of root soil consolidation were all aimed at the fracture and failure of herb roots; however there is an urgent need of studying the model of arbor roots reinforcing soil.

Considering the widely-distributed arbor roots and red clay in Hainan as the research object, this study performed a large-scale shear test on root soil complex. The anti-sliding effect of roots on soil and the law of root deformation were revealed, and the root–soil interaction mechanism was investigated. Consequently, the Wu model was improved based on the morphology and stresses of roots. The research results are expected to play an important role in the prevention and control of geological disasters under common typhoon and rainstorm weather in Hainan Province. Moreover, they can guide the prioritization of vegetation types in ecological treatment as well as slope protection and reinforcement measures after planting.

## Materials and methods

### Test device

**Figure 1:**
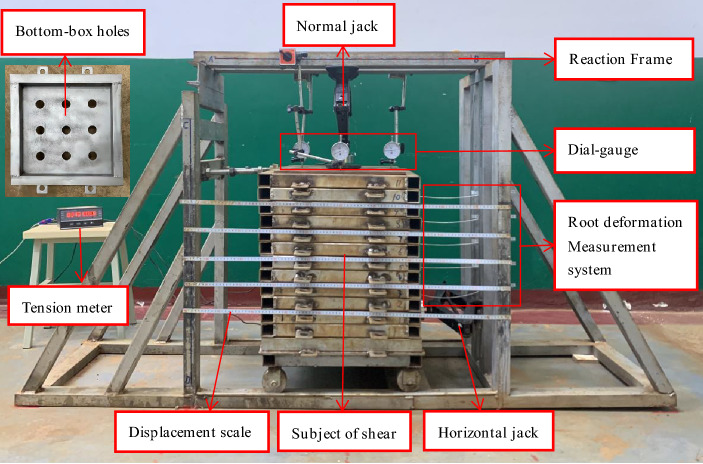
Large single shear apparatus for root–soil complex.

The adopted test device included a self-designed large single shear apparatus for the root–soil complex^43^, as shown in Fig. 1. It comprised six parts: reaction frame, shear stacking box, vertical loading system, horizontal loading system, shear displacement measurement system, and root deformation measurement system. The shear stacking box comprised 13 layers of square stacking rings with inner diameter of 40 × 40 cm and height of 4 cm. A needle of 1 mm diameter was installed between the stacking boxes to reduce friction. The top stacking box was horizontally constrained via the screw and reaction frame. Further, the bottom stacking box was set with 9-hole positions to limit the roots to different locations, and 4 rollers were welded to reduce friction. Moreover, the stress measuring device comprised a *MCK*-*S* dual-channel controller and a *JLBU*-*1* spoke type pulling pressure sensor (*Zhongwan Jinnao*) with a comprehensive measuring accuracy of 0.05%. They were used for monitoring normal pressure and horizontal thrust, respectively, where the horizontal thrust acted on the bottom 3 layers of stacked boxes to form 10 possible sliding surfaces. Further, the shear displacement of each stacking box was measured using a sticking ruler with a stainless-steel plane. In addition, the root deformation measurement system comprised a measuring rope and a measuring ruler.

### Test material

**Figure 2:**
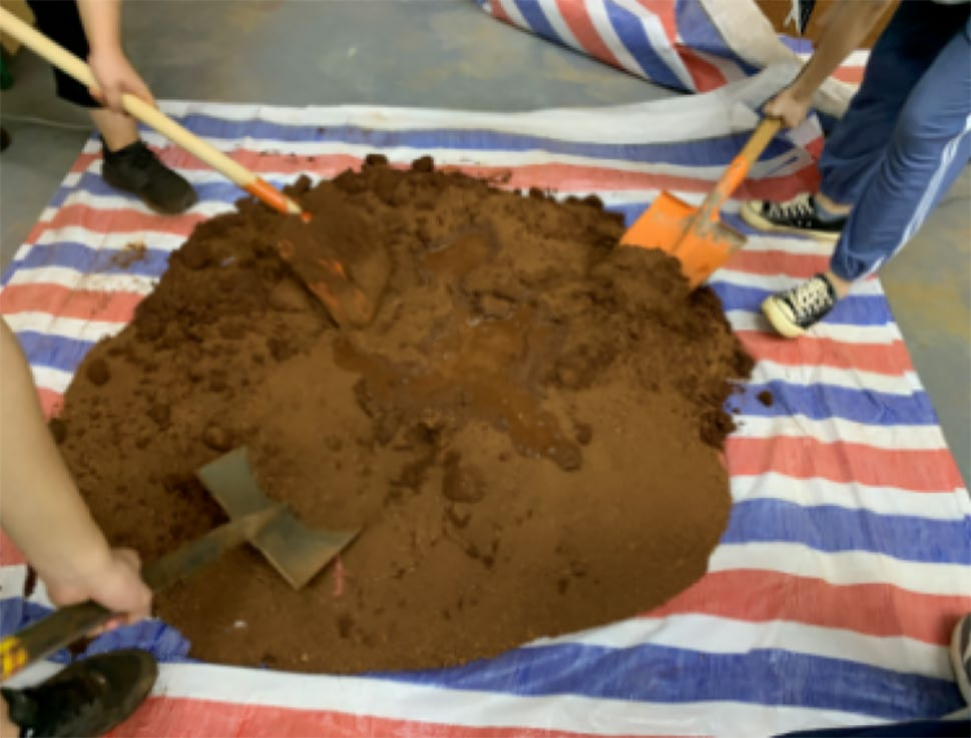
Test soil sample.

**Figure 3:**
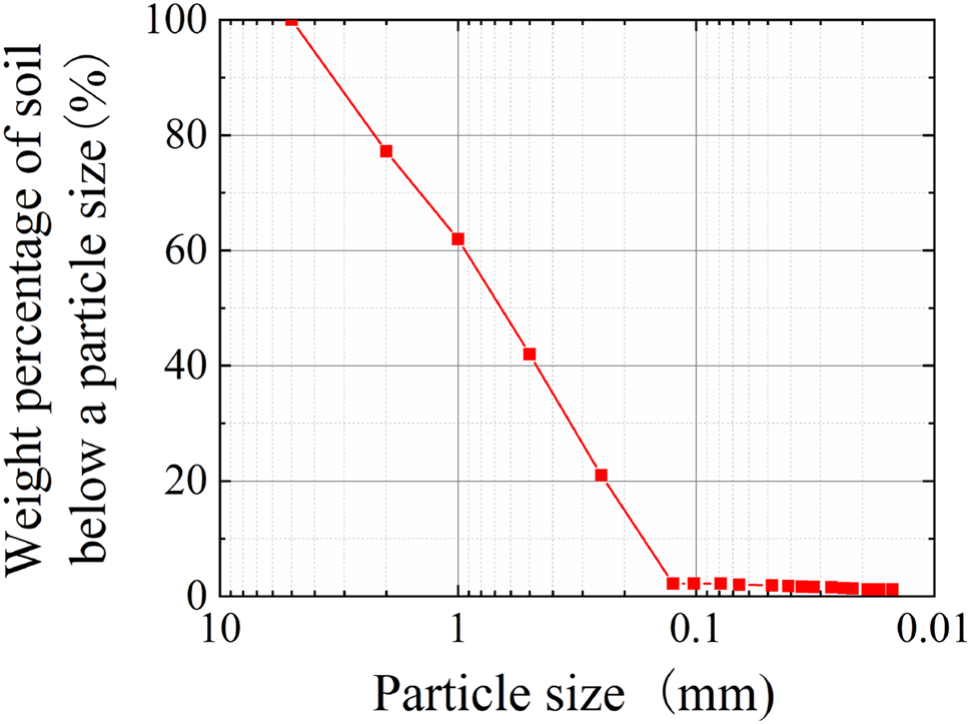
Cumulative curve of particle gradation.

Hainan is located at the edge of the tropics and experiences a tropical monsoon maritime climate. It is subject to high temperature and rain, strong weathering such as typhoons and heavy rainfall, and is prone to landslides. The test soil sample was acquired from a slope at the Guilin Yang Town, Meilan District, Haikou City. It was brick red clay, as shown in Fig. 2. At a depth of 1.5–2 m, the soil was uniform, with the particle grading curve as shown in Fig. 3. The basic physical attributes are listed in Table 1. Banyan is a representative arbor in the tropical rainforest area of Hainan, with a long life span, fast growth rate, well-developed root system, good wind resistance and environmental adaptability, and a good demonstration for the promotion of vegetation management. All the roots required in the test were obtained through reasonable field collection, and were uniform in thickness, with an average diameter of 22 mm and length of approximately 56 cm.

**Table 1:** Basic physical attributes of red clay.

Soil type	Maximum dry density *ρ*_*dmax*_ (g cm^−3^)	Optimum water content (%)	Weight *G*_*s*_	Plastic limit *ω*_*p*_ (%)	Liquid limit *ω*_*L*_ (%)
Red clay	1.46	31.2	2.7	32	64

### Test methods and steps

**Figure 4:**
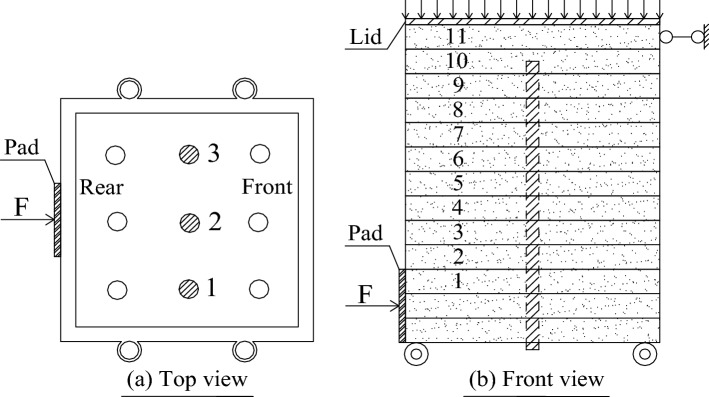
Arrangement of the root system.

**Figure 5:**
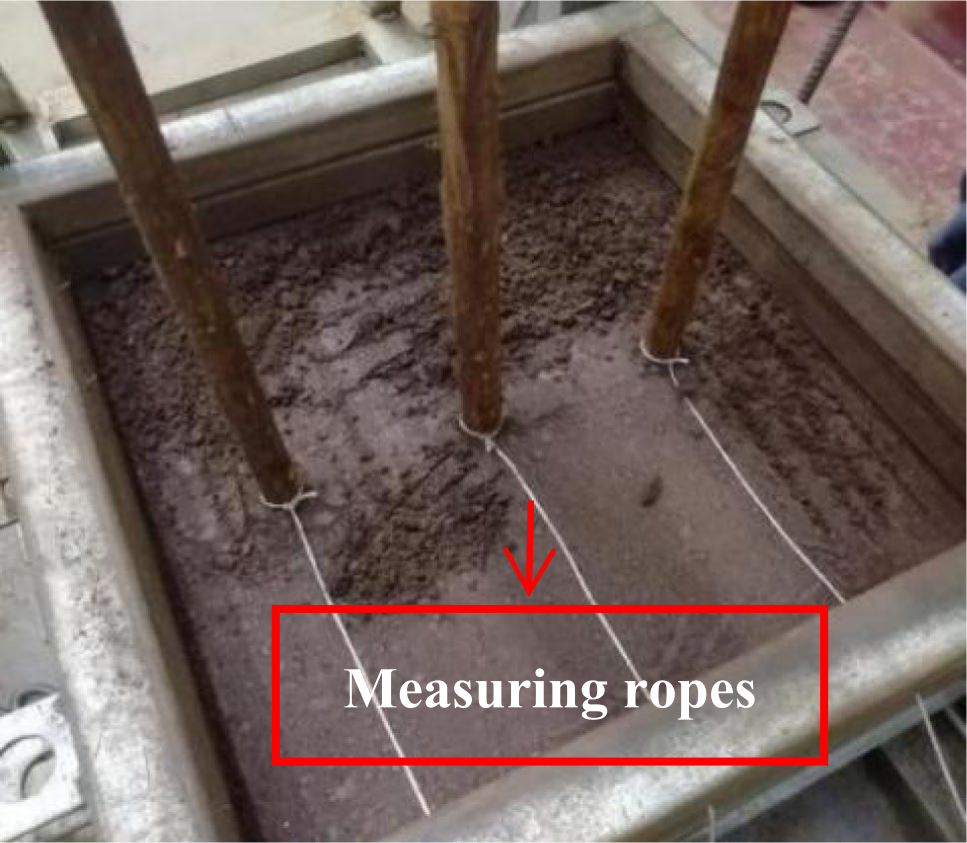
Layout of measuring ropes.

The experiment involved single shear tests of plain soil and soil complex with three parallel roots. The water content of red clay was set as 31% with the dry density being 1.06 g/cm^3^. The arrangement of the root system is shown in Fig. 4 (shaded part in the figure indicates the location of the root system). Before performing the test, the soil sample was prepared according to the preset water content and rested for one day and night. Further, prior to filling the soil sample, the root system was parallelly passed through the holes positions of the bottom stacking box into the single shear apparatus. Subsequently, the soil sample was filled and compacted six times. Thereafter, the samples were scraped from between layers with a scraper knife, wherein the measuring ropes were tied to the root system within the gaps between the stacking box numbers 1–2, 3–4, 5–6, 7–8, and 9–10, as shown in Fig. 5. It was ensured that the root system was always vertical during the filling process of the sample. Furthermore, the tests were conducted after all the soil samples were filled and rested for 12 h.

**Figure 6:**
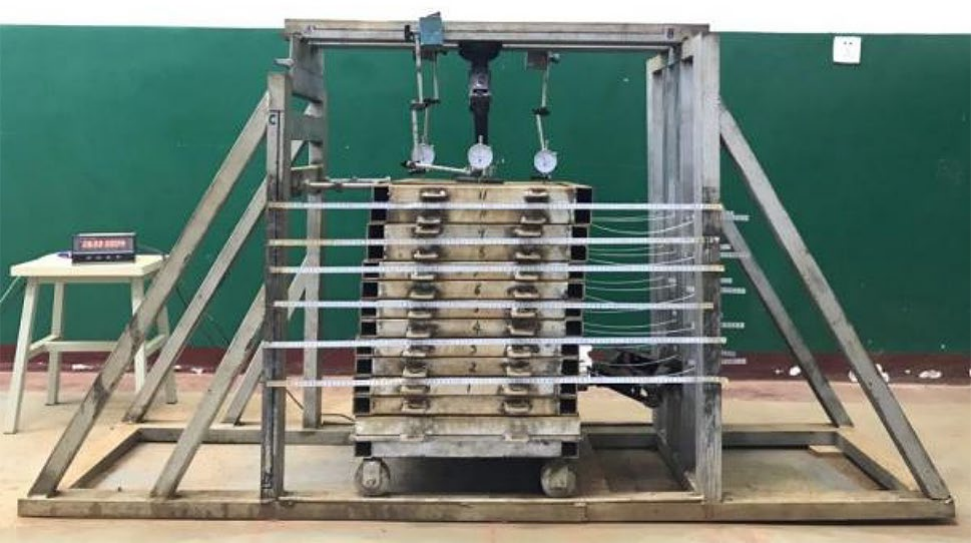
Sample after single shear failure.

Considering the stress state at the actual penetration depth of roots, the normal stress was set to 20, 40, and 60 kPa, respectively. After pressurization, according to 1, 2, 5, 10, 20, 30, and 60 min, etc., the vertical deformation reading is measured until stability. The stability standard for the specimen deformation is not greater than 0.03 mm per hour. Subsequently, the manual shear test was started and the horizontal loading speed was controlled to 1/8 circle/time. After each level of loading, the vertical deformation of soil, horizontal displacement of 11-layer stacking boxes, root deformation and horizontal thrust were recorded. The entire test required approximately 2 h when the displacement reached 60 mm. As per the Standard for Geotechnical Testing Method^44^, the shear force at stress peak or a displacement of 40 mm (10% of the stacking box length) was considered as the shear strength. Consequently, the gradient surface emerged during shear failure, as shown in Fig. 6.

## Results analysis and discussion

### Strengthening effect of root system on soil

**Figure 7:**
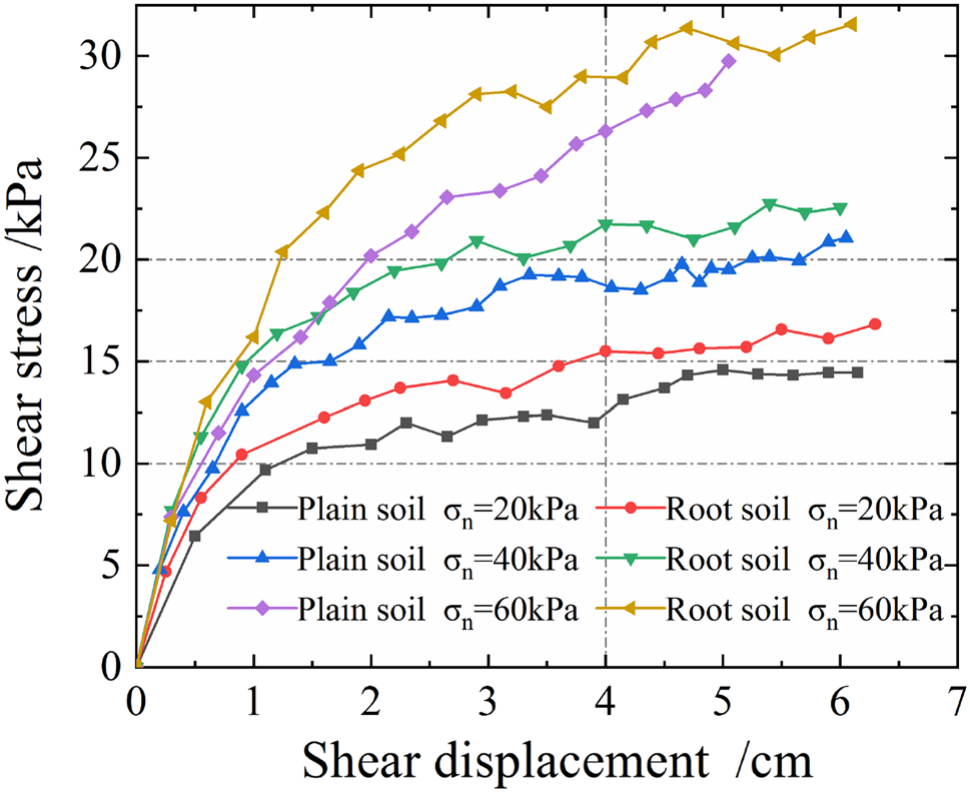
Relationship curve between shear stresses and shear displacements.

Figure 7 shows the relationship curve between the shear stresses and shear displacements of plain soil and soil complex with three parallel roots under different normal stresses. Herein, the soil complex with three parallel roots was referred to as root soil.

As evident from Fig. 7, the shear stresses of plain and root soils both grew with the increase in their shear displacements, and both exhibited the characteristics of strain hardening type. This was because there was no artificially set surface of shear failure for single shearing. The sheared soil sample experienced a gradual failure process, and the shear strength of the root soil was greater than that of the soil sample itself. Further, The shear failure often occurred within a certain range of the soil; therefore, the shear stress grew with the increase in shear displacement. Moreover, under the same normal stress state, the shear stress of the root soil was significantly higher than that of plain soil, indicating that the arbor roots significantly improved the shear strength of soil and resulted in its consolidation.

The reinforcing effect of the root system on the slope was analyzed according to the root content in the soil. The calculation formula of root area ratio^45^ is expressed as:

1 $$RAR = \frac{{A_{r} }}{{A_{s} }} = \frac{{\sum\nolimits_{i = 1}^{n} {\pi d_{i}^{2} /4} }}{l \cdot b} \times 100\%$$

where *RAR* is the root area ratio (%), *Ar* is the sum of all roots' cross-sectional areas, *As* is the cross-sectional area of the sample, *d*_*i*_ is the diameter of a single root, *l* and *b* are the length and width of the single shear apparatus, respectively, and *n* is the number of roots.

**Figure 8:**
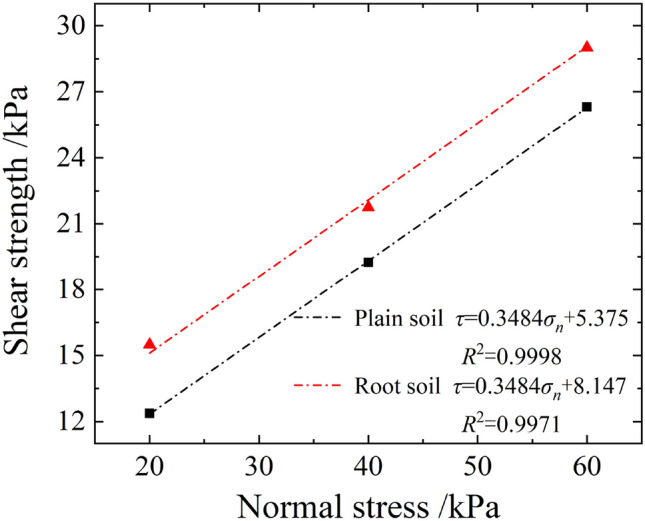
Shear strength curve.

The Wu model^29–31^ assumes that the reinforcing effect of plant roots on soil is mainly reflected through the increase in cohesion, while the influence on the internal friction angle is minimal. According to this calculation, the shear strength indices of plain and root soils are listed in Table 2, and the shear strength curve is shown in Fig. 8. As evident, the cohesion of root soil (*RAR* = 0.71%) was significantly higher than that of plain soil, reaching 51.58%.

**Table 2:** Shear strength indices of plain and root soils.

Sample type	Root area ratio *RAR*/%	Internal friction angle *φ*/°	Cohesion *c*/kPa	Cohesion growth $\Delta c$/kPa	Cohesion growth rate $\Delta c/c_{plain}$/%
Plain soil	0	19.21	5.38	2.77	51.58
Root soil	0.71		8.15

**Figure 9:**
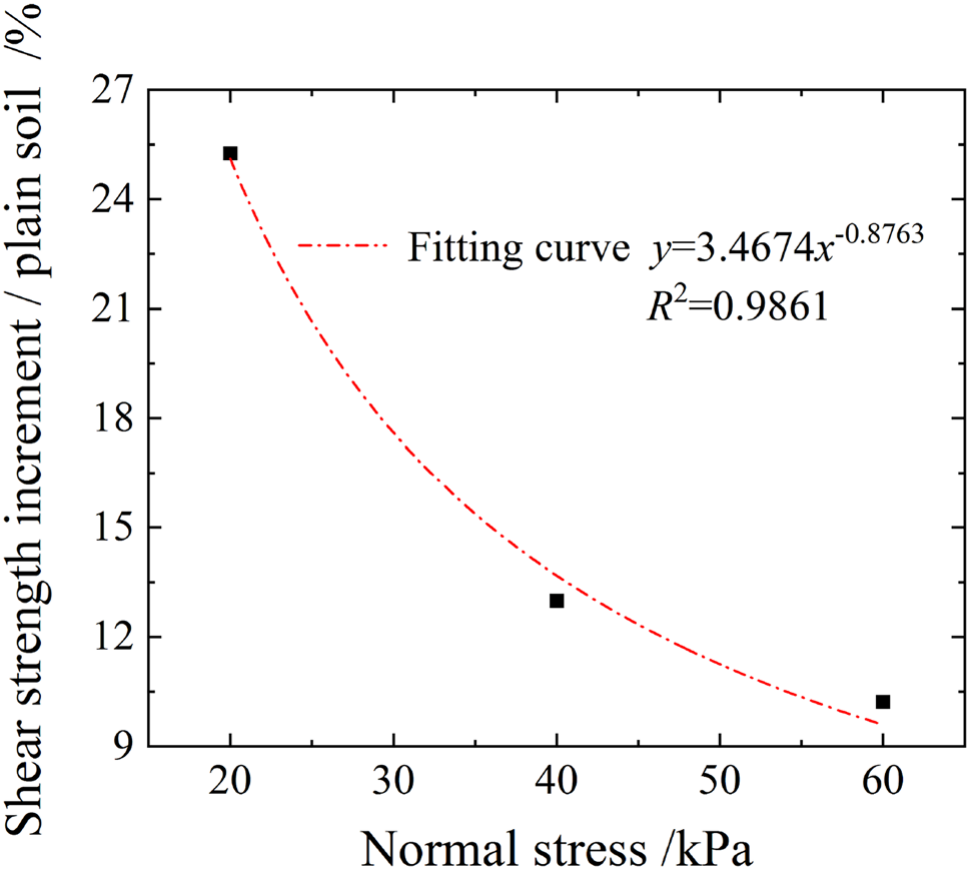
Shear strength growth curve.

After sorting the shear strength growth under each normal stress state, as shown in Fig. 9, the shear strength growth was found to decrease as per the power-law with an increase in normal stress. When the normal stress increased from 20 to 60 kPa, the shear strength growth decreased from 25.3 to 10.2%. Moreover, in case of a shallow landslide, the soil consolidation effect of root system was obvious. However, with the increase in depth, the soil shear strength owing to self-weight consolidation also increased, whereas the strengthening effect of the root system on soil gradually decreased.

### Anti-sliding effect of root system on soil

**Figure 10:**
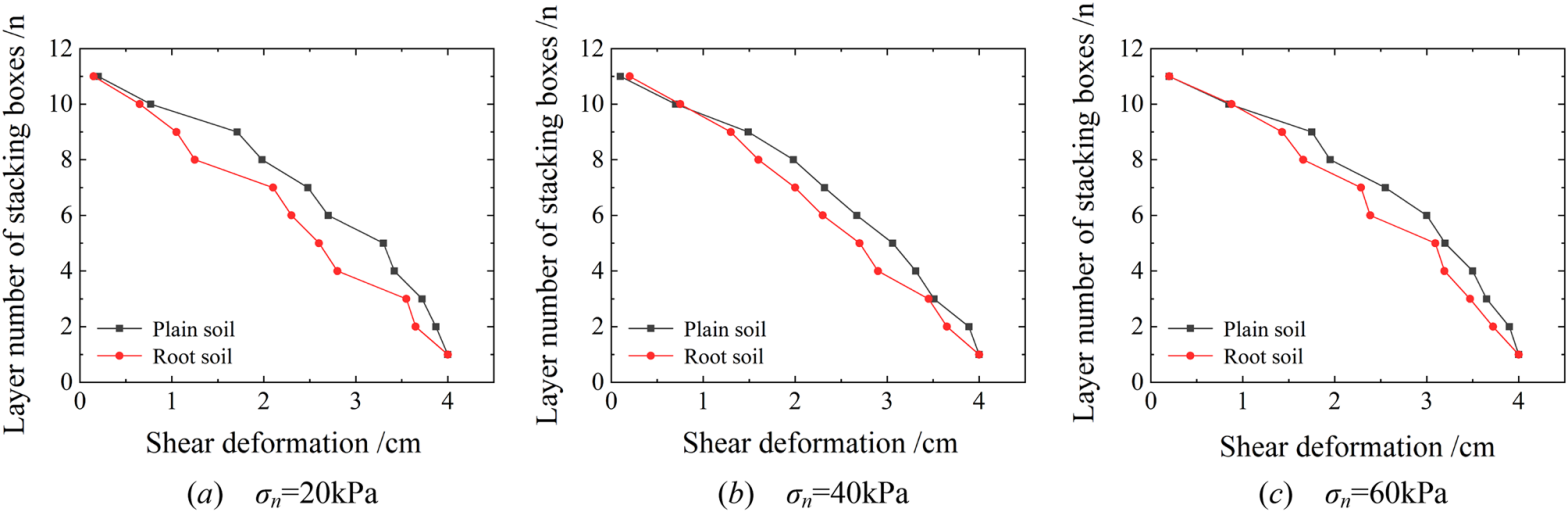
Shear deformation patterns of each stacking box.

For a shear displacement of 4 cm, the shear deformation patterns of plain and root soil samples obtained under different normal stresses are as shown in Fig. 10.

As evident, the shear displacement reached the maximum at the place where the shear stress exerted its effect. Moreover, with increase in the distance from the acting surface of shear stress, the shear displacement reduced. Under the same normal stress, the shear deformations at different heights of the root soil sample were all significantly smaller than those of the plain soil. However, the difference between them gradually reduced with increase in the normal stress, indicating that the anti-sliding effect of the root system on the soil gradually decreased with the increase in normal stress.

Table 3 lists the shear displacements of the bottom stacking box under the action of typical normal and shear stresses in the shear process extracted from Fig. 7. The shear deformation of the root soil was generally 37.5% lower than that of the plain soil. This demonstrated the ability of the arbor roots in effectively improving the ability of the slope to resist deformation and delay the occurrence of landslides.

**Table 3:** Shear displacements of the bottom stacking box under typical normal and shear stresses.

Sample type	*σ*_*n*_ = 20 kPa	*σ*_*n*_ = 40 kPa	*σ*_*n*_ = 60 kPa
	*τ* = 10 kPa	*τ* = 15 kPa	*τ* = 20 kPa
Plain soil	1.22 cm	1.65 cm	1.97 cm
Root soil	0.83 cm	0.95 cm	1.23 cm
Decrease	32.0%	42.7%	37.7%

### Deformation pattern of root system

**Figure 11:**
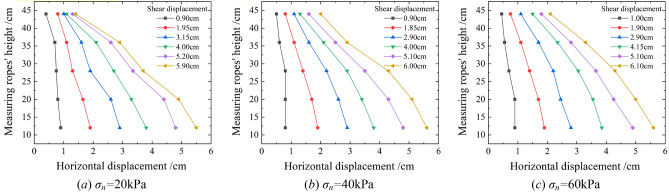
Root deformations in shearing under different normal stresses (Root No. 1).

Root deformation in shearing is crucial to the consolidation performance of soil, which is the premise of studying the mechanism of root–soil interaction. As the shear deformation patterns of root Nos. 1, 2, and 3 were the same, Fig. 11 only presents the results of root No. 1 under different normal stresses.

**Figure 12:**
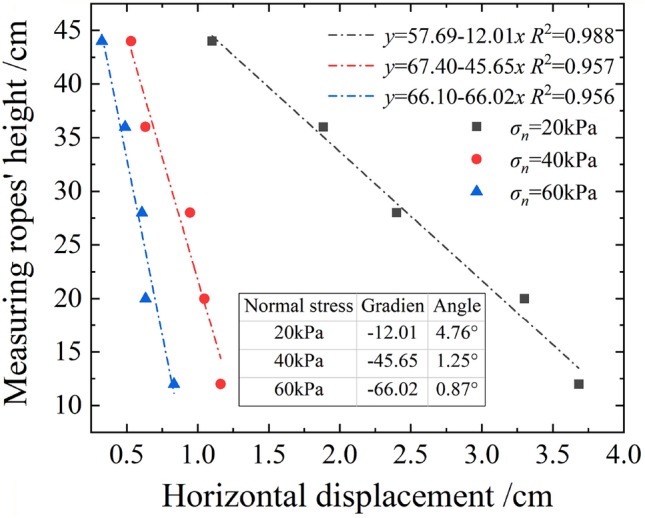
Root deformations under different normal stresses when *τ* = 15 kPa (Root No.3).

As evident, the root deformation gradually increased with the growth in shear displacement, and the root morphology gradually varied from a straight line into a curve. For shear stress *τ* = 15 kPa, the root deformation decreased gradually with the increase in normal stress. When the normal stress increased from 20 to 60 kPa, the root deformation angle decreased by 82%, as shown in Fig. 12. This confirmed that the high-stress state was not conducive to inducing the soil-retaining effect of the root system.

**Figure 13:**
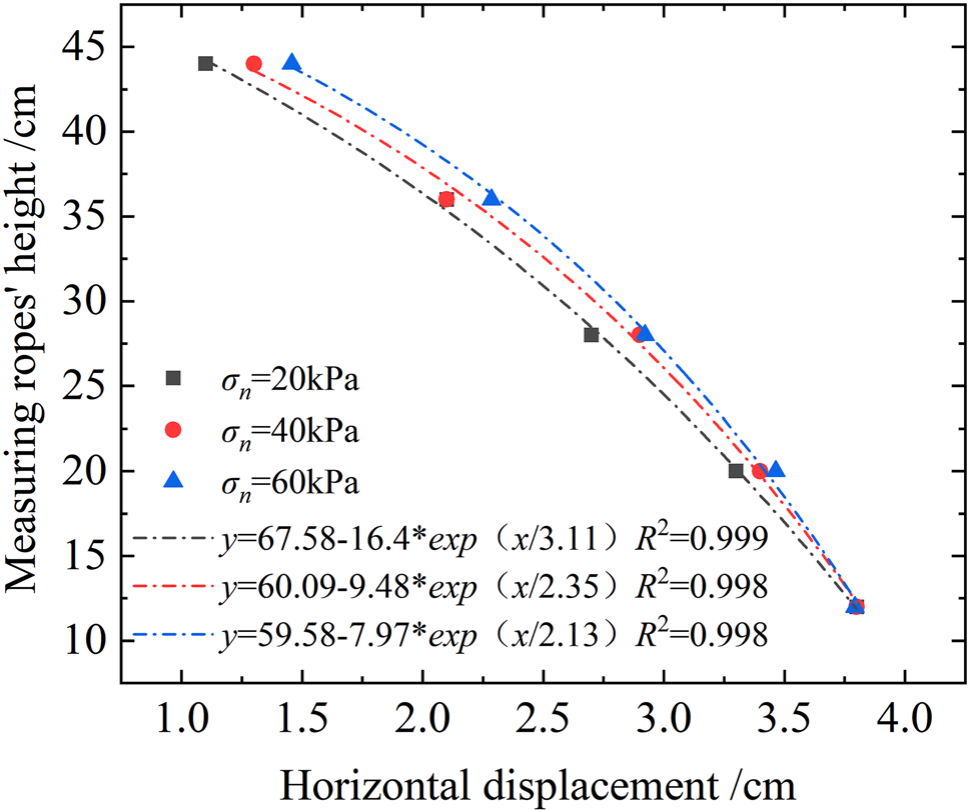
Root deformations of shear failures under different normal stresses (Root No.1).

Figure 13 shows the root deformation patterns under different normal stress states when the shear displacement was 4 cm (Root No.1). As evident, the root morphology in the shear failure state was a convex curve, which could be described by $y = y_{0} + A \cdot \exp (x/t)$. The root convex deformation increased gradually from the bottom to the top with the growth in normal stress, implying that the soil-retaining effect of the upper part of the root system became increasingly prominent with the increase in normal stress. In the test, the bottom of the root system was constrained in the horizontal direction, whereas the top was affected by the soil present around the root. With the increase in normal stress, the ultimate shear stress increased, and the movement of soil particles in the stacking box intensified. Owing to the soil-retaining effect of the bottom of the tree roots, the upward movement of soil particles was strengthened; therefore, the differential deformation of the upper root system became gradually prominent.

### Root–soil interaction mechanism

Based on the results of the normal and horizontal loading tests, the influences of soil particle movement and root deformation on the anti-sliding effect were explored to reveal the mechanism of root–soil interaction.

**Figure 14:**
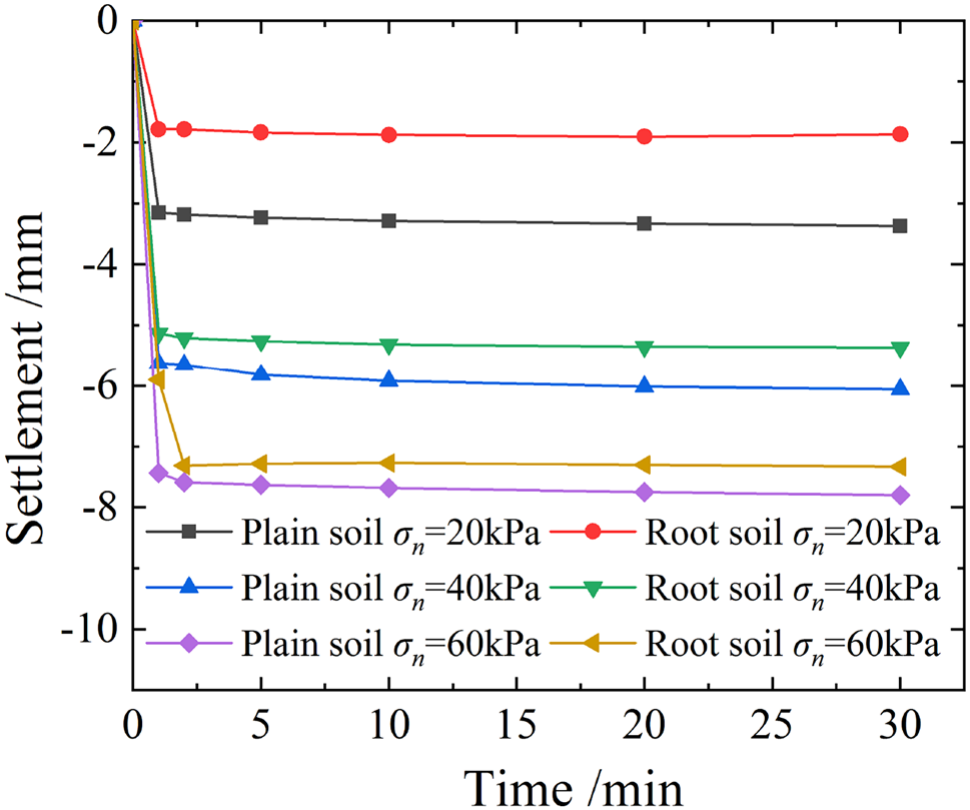
Curves of settlement varying with time under different normal stresses.

**Figure 15:**
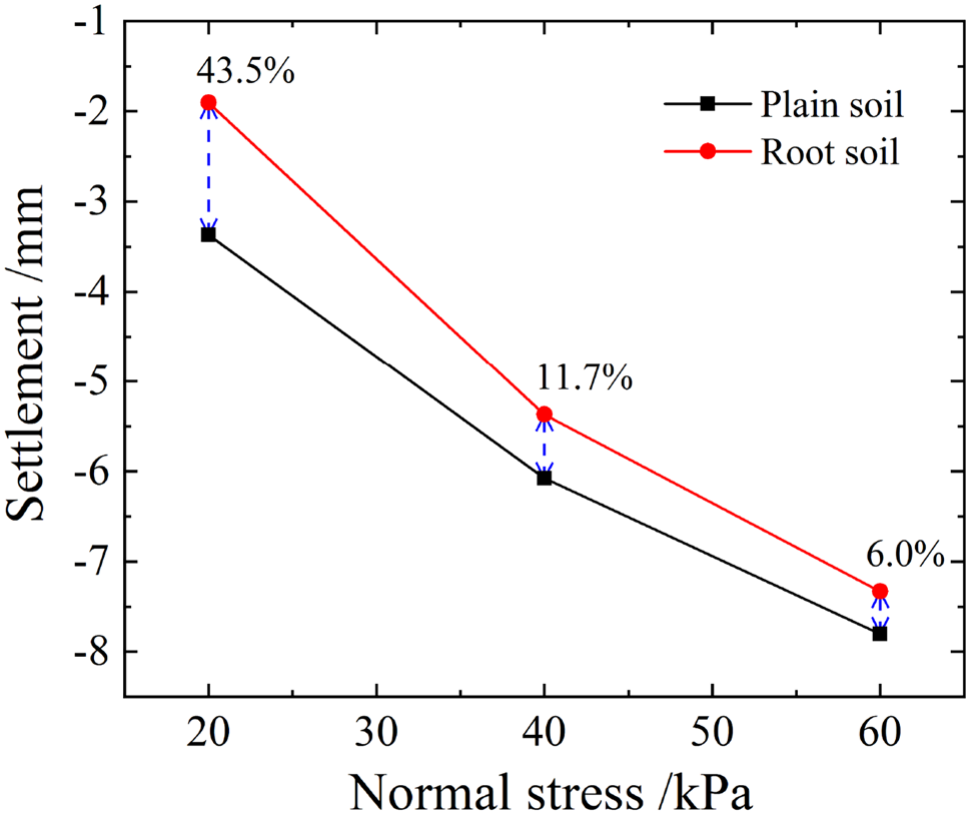
Relationship curve between stable settlements and normal stresses of plain and root soils.

Figure 14 shows the settlement patterns of plain and root soil samples during normal loadings. As evident, the settlement deformation under different normal stresses was primarily an instantaneous settlement, which basically tended to stabilize within 30 min. Under same state of normal stress, the stable settlement of plain soil was greater than that of root soil; however, the gap between them gradually decreased with the increase in normal stress, as shown in Fig. 15. This was because the existence of tree roots increased the overall stiffness of soil and improved its compressive bearing capacity.

**Figure 16:**
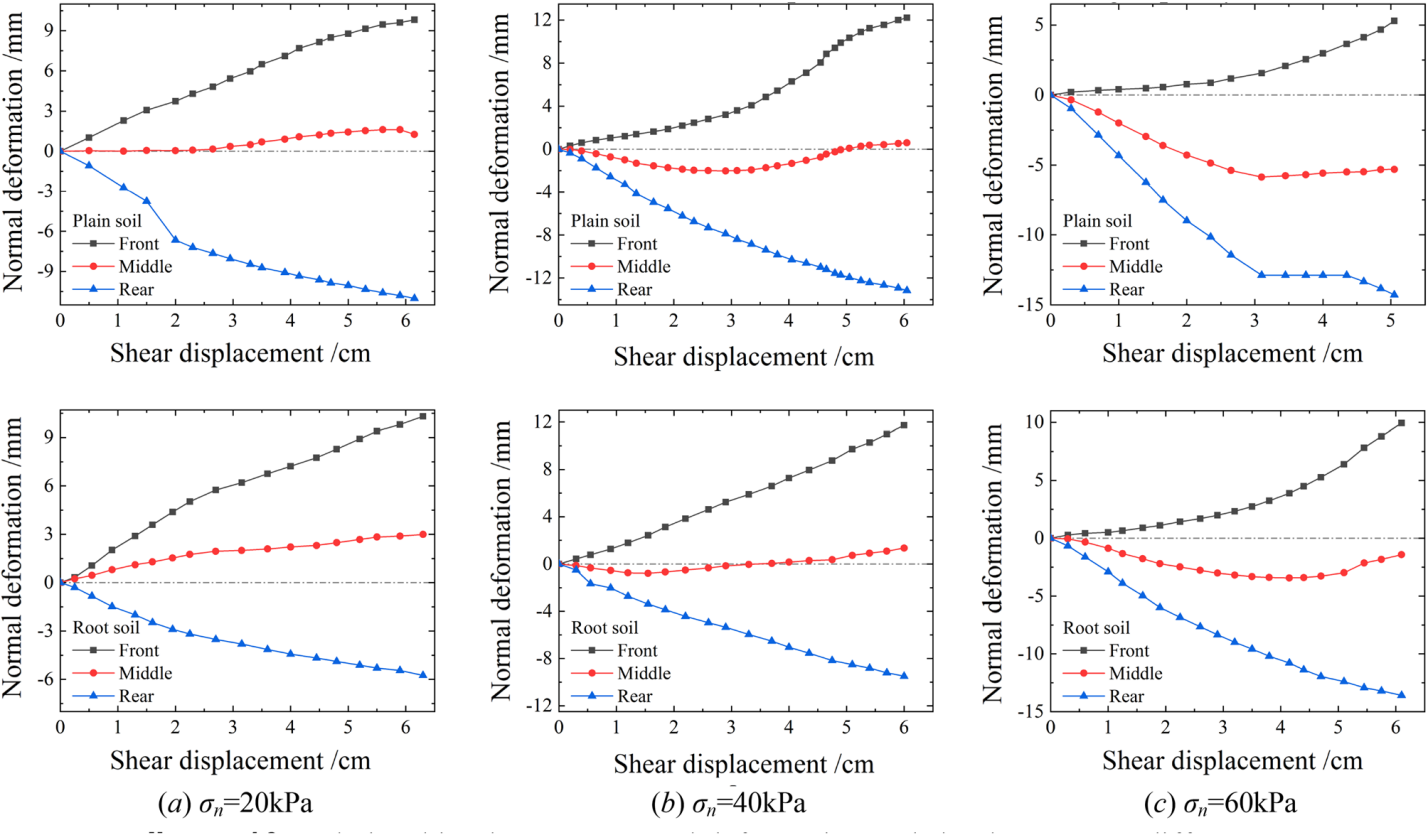
Relationship curve between normal deformation and shear displacement at different parts.

Figure 16 shows the normal deformation patterns of different parts on the surfaces of plain and root soils during shearing. The soil particles exhibited two motion states: translation and rotation. Under the action of horizontal thrust, the soil particles at the back of the stacking box moved forward, and those at the front accumulated owing to boundary constraints under the promotion of horizontal shear stresses and the presence of soil particles at the back. With the gradual increase in shear stress, the soil particles at the front inevitably moved upward after squeezing and rolling each other, thus exhibiting the phenomenon of being higher at the front and lower at the back.

**Figure 17:**
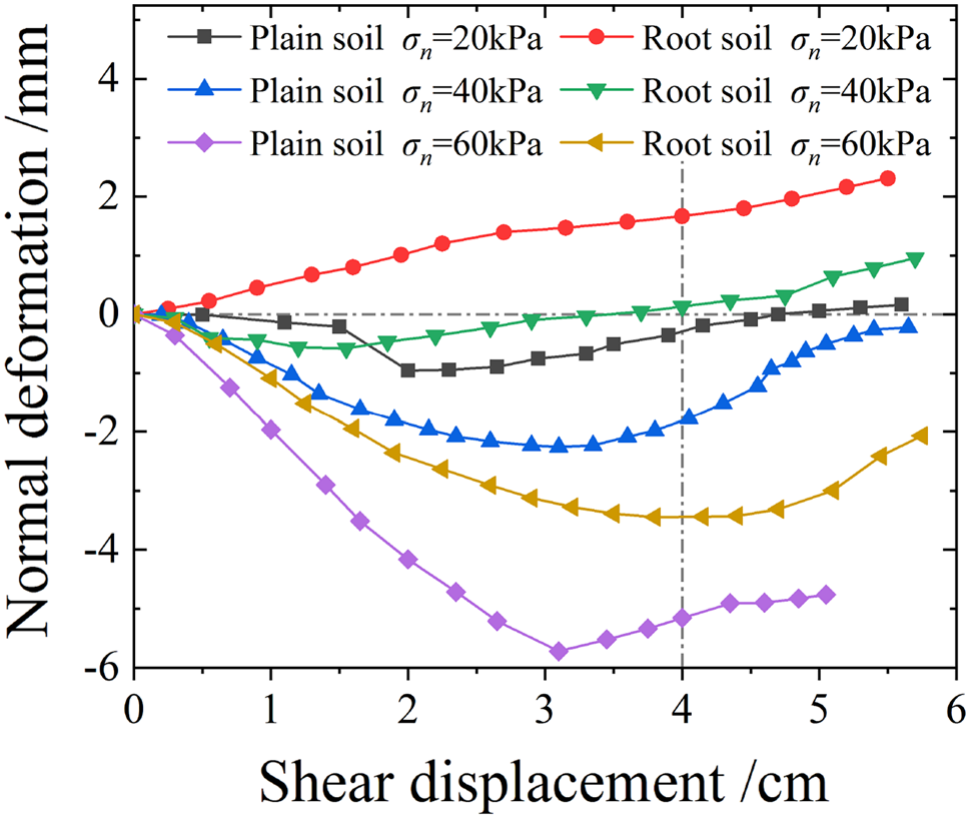
Relationship curve between the normal deformations' average values and the shear displacements of plain and root soils.

**Figure 18:**
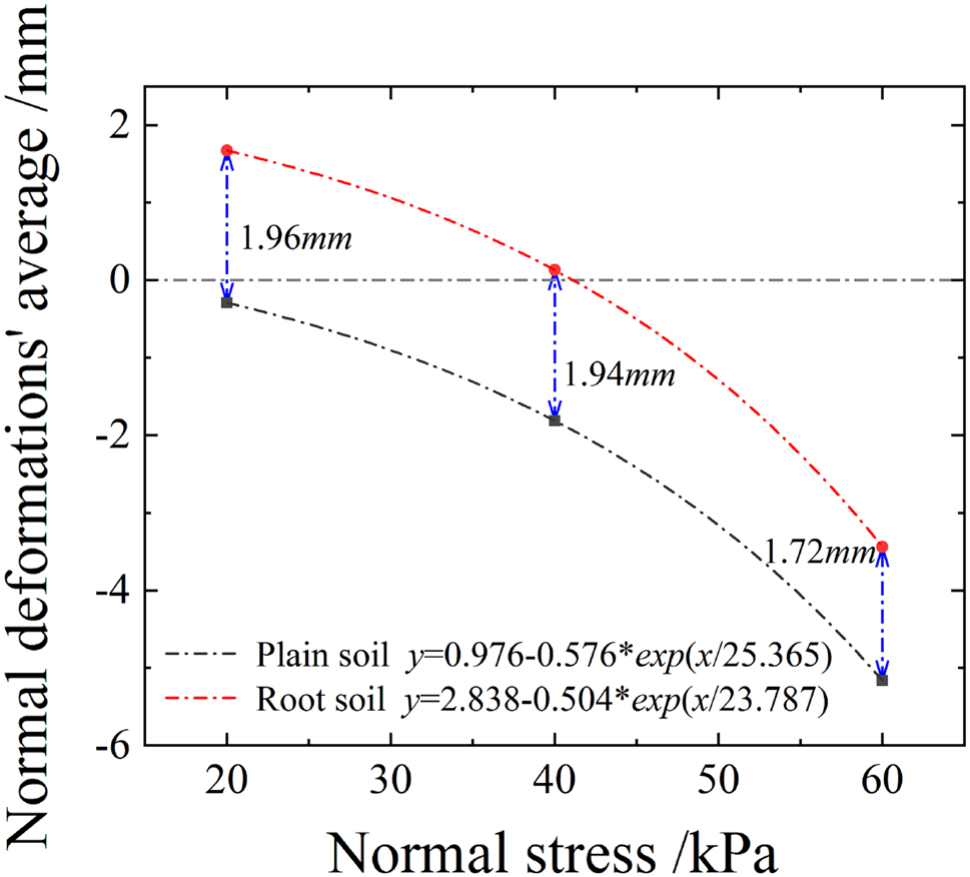
Relationship curve between normal deformations' average values and normal stresses of plain and root soils (shear displacement of 4 cm).

Figure 17 shows the relationship curve between the normal deformations' average values and the shear displacements of plain and root soils under different normal stresses during the shear process. As evident, under the joint action of the normal and horizontal forces, plain soil exhibited the shear shrinkage effect, which intensified with the increase in normal stress. For *σ*_*n*_ = 20 or 40 kPa, the shear expansion effect was observed, whereas for *σ*_*n*_ = 60 kPa, the shear shrinkage effect occurred. For shear displacement of 4 cm, the settlement of root soil was below that of plain soil, and the gap between these gradually decreased with the increase in normal stress, as shown in Fig. 18. This indicated that the existence of roots was conducive to the soil resisting shear settlement, and the effect was more significant under the state of low stress.

**Figure 19:**
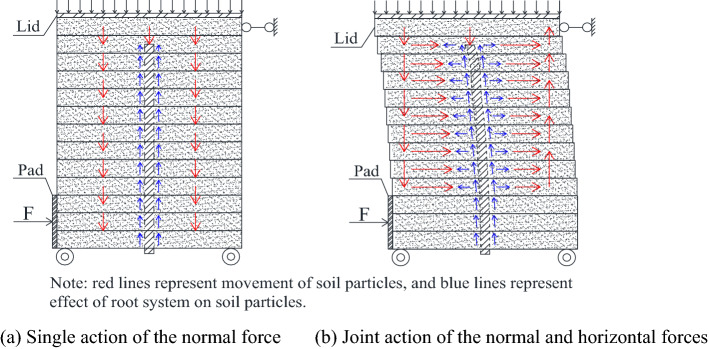
Movement diagram of soil particles.

Because the single shear test was not limited to the shear failure surface, there were certain movements of soil particles at the shear surface and inside the soil samples as follows: translation and rotation. For plain soil, the soil particles mainly moved freely under the action of shear stress, and the pores inside the soil sample were effectively compressed, thereby leading to the shear shrinkage effect. In contrast, for the root soil, the root system was subject to bending deformation under shear. It exhibited the stress states of tension and compression at the front and the rear, respectively, which resulted in the imposition of friction and pushing effects on soil particles, as shown in Fig. 19a,b. In the process of shearing, when the soil particles moved near the root system, it acted as the soil-retaining barrier, which restricted the translation of soil particles and forced them to rotate. Under low normal stress, the restriction of the root friction to the soil particles rotation was limited; hence, their upward movement exhibited the effect of shear expansion. However, under high normal stress, with the increases in the root deformation and ultimate shear stress, the root friction effectively restricted the rotation of soil particles around the root and forced them to pass through the root gaps, which resulted in the phenomenon of "flow around". Consequently, the compression and compaction of soil for the shear shrinkage effect was observed.

Thus, the essence of the interaction between arbor roots and soil lies in the movement of soil particles and the deformation of roots. Under the joint action of normal and horizontal forces, the rear soil particles promoted root deformation and movement of the front soil particles, whereas the root reacted on the rear soil particles while exhibiting the effects of friction and retaining. Moreover, when the consolidation effect at the end of the root system was insufficient, the anti-bending capacity of the root system significantly influenced the anti-sliding effect on the soil.

## Improved mechanical model of root system consolidating soil

The Wu model is the first mechanical model to propose the soil consolidation effect of plant roots, which can be used to calculate the contribution of plant roots to the shear strength of soil. Because of its clear principle and simple calculation, it remains the most applied model for evaluating soil consolidation by roots. Its mechanical equations are expressed as follows:

2 $$\tau_{r} = \tan \phi \cdot \sigma_{n} + c + \Delta c$$

3 $$\Delta c = \sum\limits_{i = 1}^{m} {T_{ri} } \cdot RAR_{i} \cdot k = \sum\limits_{i = 1}^{m} {T_{ri} } \cdot RAR_{i} \cdot (\cos \theta + \sin \theta \tan \phi )$$

where *τ*_*r*_ is the shear strength, *σ*_*n*_ is the normal stress, *φ* is the internal friction angle of plain soil, *c* is the cohesion of plain soil, *Δc* is the shear resistance provided by root system, *T*_*ri*_ is the ultimate tensile strength of the single root, *RAR*_*i*_ is the root area ratio of single root, *θ* is the offset angle of root system after shear, and *k* is the contribution coefficient of root strengthening, which can be used to evaluate the reinforcement and consolidation effects of the root system on soil.

**Figure 20:**
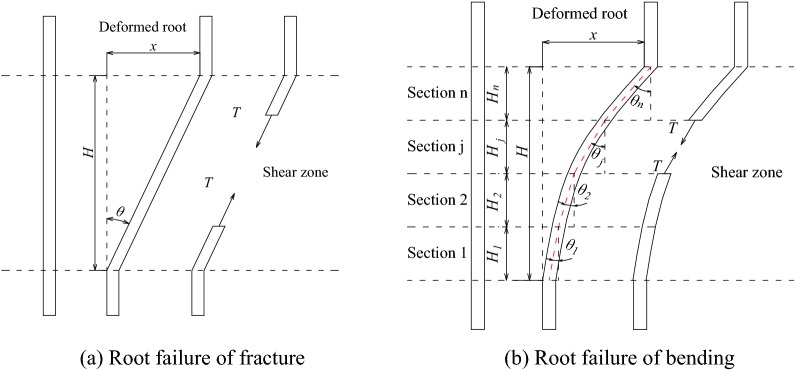
Stress diagram of different modes of root failures.

The Wu model hypothesizes that the root ends are not pulled out and that all roots break simultaneously when reaching the maximum tensile strength (Fig. 20a), which is an obvious overestimation of the contribution of roots to the shear strength of soil. Meanwhile, in case of the arbor root system with strong root rhizomes, there are three breaking modes in shear failure: fracture, pullout, and slip^42^; thus, the Wu model is apparently no longer applicable.

In order to evaluate the mechanical effect of arbor roots in consolidating soil more reasonably, the Wu model was improved based on the damage morphology and pattern of arbor roots under shear failure. On the one hand, when the shear failure of the root–soil complex occurs, the soil reaches the limit state, while the root system bends and slips. Therefore, the root's tensile strength exerting coefficient *α*_*i*_ is introduced to define the actual stress state of the root system as shown in Eq. (4). On the other hand, according to the measured results, the actual root deformation is curved, and the deformation increment of each segment is inconsistent. Therefore, the convex curve of arbor root system is divided into multiple straight lines based on the idea of calculating and superposition of curve segments, as shown in Fig. 20b. Then the improved contribution coefficient of root strengthening *kʹ* was calculated, as shown in Eq. (5). Ultimately, the improved cohesion increment in the Wu model is as follows Eq. (6).

4 $$T^{\prime}_{ri} = \alpha_{i} T_{ri}$$

5 $$k^{\prime} = \sum\limits_{j = 1}^{n} {\frac{{(\cos \theta_{j} + \sin \theta_{j} \tan \phi )H_{j} }}{H}}$$

6 $$\Delta c = \sum\limits_{i = 1}^{m} {T^{\prime}_{ri} } \cdot RAR{}_{i} \cdot k^{\prime} = \sum\limits_{i = 1}^{m} {\alpha_{i} T_{ri} } \cdot RAR{}_{i} \cdot \sum\limits_{j = 1}^{n} {\frac{{(\cos \theta_{j} + \sin \theta_{j} \tan \phi )H_{j} }}{H}}$$

where $T^{\prime}_{ri}$ is the single root's tensile stress, *H* is the height of the shear zone, *H*_*j*_ is the height of shear zone of the section *j*, and *α*_*i*_ is the root's tensile strength exerting coefficient, obtained through tests or experience.

Because the root system deformation patterns of root Nos. 1, 2, and 3 were the same, this study considered the deformation of root No. 1 to calculate both the root-strengthening contribution coefficients and the average tensile strengths of roots before and after the improvement of Wu model. The specific calculation process was presented in Table 4. As evident from the calculations, the tensile strength of the root system was only 4% effective under the shear failure state (for shear displacement of 4 cm). This is because neither the upper nor the lower ends of the root system had been anchored in the test, with only the restrictions from soil around it being considered. The improvement of the shear strength mainly originated from the anti-bending stiffness of the root system. Therefore, it was suggested that the embedded depth of the root system should be increased in the actual project to improve the anchoring effect of the root system and exploit its tensile strength. The improved contribution coefficient of root strengthening *kʹ* was less than *k*, which decreased gradually with the increase in normal stress. Again, this proved that the increase in normal stress weakened the soil consolidation effect of root system. Because the preset shear deformation of root system in this test was only approximately 5°, the general value of root strengthening contribution coefficient was relatively small, whereas the variation before and after the improvement was insignificant. However, the increased cohesion of the roots calculated using Eq. (3) remained significantly higher than the actual case. Thus, the improved model better reflected the actual stress state and deformation of roots. Further, it deepened the research on the mechanical model of plant roots consolidating soil, and provide a theoretical basis for better prediction and evaluation of the soil consolidation effect of roots.

**Table 4:** Calculation of root reinforcement contribution coefficient (Root 1).

Normal stress (kPa)	Measuring ropes' height (cm)	Root deformation (cm)	Root offset angle *θ*_*j*_ (°)	Section j's contribution coefficient *k*_*j*_	Accumulated contribution coefficient *kʹ*	Single root's average tensile stress (kPa)	Average exerting coefficient *α*	Linear offset angle *θ* (°)	contribution coefficient *k*
20	12	3.8			1.0254	427.56	4.61%	4.8067	1.0257
			3.5763	1.0198					
	20	3.3							
			4.2892	1.0233					
	28	2.7							
			4.2892	1.0233					
	36	2.1							
			7.125	1.0355					
	44	1.1							
40	12	3.8			1.0238	342.59	3.70%	4.6055	1.0247
			2.8624	1.0162					
	20	3.4							
			3.5763	1.0198					
	28	2.9							
			5.7106	1.0297					
	36	2.1							
			5.7106	1.0297					
	44	1.3							
60	12	3.79			1.0224	368.79	3.98%	4.2727	1.0232
			2.3315	1.0133					
	20	3.46							
			3.882	1.0213					
	28	2.92							
			4.5434	1.0245					
	36	2.29							
			5.9131	1.0306					
	44	1.46							

The ultimate tensile strength of arbor taproot is expressed as *T*_*m*_ = 531.76*d*^−1.31^, where *T*_*m*_ unit is MPa, and the diameter unit is mm^46^. So the single root's ultimate tensile strength is 9.27 MPa in the test.

## Conclusions and prospects

This study considered the typical red clay and arbor roots at Hainan as the research object and conducted shear tests on plain soil and soil complex with three parallel roots under different normal stresses using a self-developed large-scale instrument of single shear for root soil complex. The primary conclusions of this study are as follows:

- The arbor roots exhibited a significant reinforcing effect on shear strength and ductility of soil. Further, the cohesion of the root soil complex (RAR = 0.71%) increased by approximately 50% and its ductility increased by approximately 37.5% compared to that of plain soil.
- The volumetric strain of plain soil and root–soil complex under shear failure exhibited different performances. With the increase in normal stress, the plain soil exhibited the shear shrinkage effect, whereas the root soil complex changed from the shear expansion effect to the shear shrinkage effect.
- Banyan roots in the red clay with lower stress state exerted a larger reinforcing effect than higher stress, which is beneficial for shallow ecological slope protection.
- The movement of soil particles in the root–soil complex during the shear process resulted in root deformation, and the effect of the roots on the soil particles was manifested as the friction and barrier effect. Upon the occurrence of shear failure, the soil mass failed in the shear mode, whereas the root system bent and slipped.
- The arbor root morphology in the shear failure state could be described by $y = y_{0} + A \cdot \exp (x/t)$. The improved Wu model was found to better reflect the stress state and deformation of root system.

This study creatively disclosed the deformation pattern of arbor roots during the shear process, which provides an experimental and theoretical basis for the establishment and improvement in the mechanical model of arbor roots consolidating soil. However, the determination method of the root’s tensile strength exerting coefficient *α*_*i*_ must be further investigated, and research on the interaction between the roots is still lacking.

## Data Availability

The datasets collected and analyzed during the current study are available from the corresponding author on reasonable request.
